# Traditional Uses, Phytochemistry, and Pharmacology of *Olea europaea* (Olive)

**DOI:** 10.1155/2015/541591

**Published:** 2015-02-23

**Authors:** Muhammad Ali Hashmi, Afsar Khan, Muhammad Hanif, Umar Farooq, Shagufta Perveen

**Affiliations:** ^1^Department of Chemistry, COMSATS Institute of Information Technology, Abbottabad 22060, Pakistan; ^2^Department of Pharmacognosy, College of Pharmacy, King Saud University, P.O. Box 2457, Riyadh 11451, Saudi Arabia

## Abstract

*Aim of the Review.* To grasp the fragmented information available on the botany, traditional uses, phytochemistry, pharmacology, and toxicology of *Olea europaea* to explore its therapeutic potential and future research opportunities. *Material and Methods.* All the available information on *O. europaea* was collected via electronic search (using Pubmed, Scirus, Google Scholar, and Web of Science) and a library search. *Results.* Ethnomedical uses of *O. europaea* are recorded throughout the world where it has been used to treat various ailments. Phytochemical research had led to the isolation of flavonoids, secoiridoids, iridoids, flavanones, biophenols, triterpenes, benzoic acid derivatives, isochromans, and other classes of secondary metabolites from *O. europaea*. The plant materials and isolated components have shown a wide spectrum of *in vitro* and *in vivo* pharmacological activities like antidiabetic, anticonvulsant, antioxidant, anti-inflammatory, immunomodulatory, analgesic, antimicrobial, antiviral, antihypertensive, anticancer, antihyperglycemic, antinociceptive, gastroprotective, and wound healing activities. *Conclusions. O. europaea* emerged as a good source of traditional medicine for the treatment of various ailments. The outcomes of phytochemical and pharmacological studies reported in this review will further expand its existing therapeutic potential and provide a convincing support to its future clinical use in modern medicine.

## 1. Introduction

Oleaceae family or the family of dicotyledons includes 30 genera [1, 2] of deciduous trees and shrubs including olive tree and its relatives numbering about 600 species [3]. The family is divided into several tribes, that is, Fontanesieae, Forsythieae, Jasmineae, Myxopyreae, and Oleeae [4, 5]. These are mostly native of all continents except the Antarctic, including tropical, subtropical, and temperate regions of the world [1, 6]. Oleaceae is best grown in Asia and Malaysia especially tropical and temperate regions of Asia [7].

The genus* Olea* got its name from the Greek “elaia” and the Latin “oleum,” but it is known by nearly 80 different names (Table 1) [8]. The genus* Olea* comprises 30 species [9] but* Olea europaea* L. is the most popular member of the genus* Olea* [10]. It is the only species of this genus which is used as food [11] and is found in the Mediterranean region [12].

Olive trees are normally distributed in the coastal areas of the eastern Mediterranean basin, the contiguous coastal areas of southeastern Europe, northern Iran at the south end of the Caspian Sea, western Asia, and northern Africa [13]. Olive tree and its fruit are also important in context of religion. Olives are narrated several times in the Bible, both in the New and Old Testaments [13]. Olive has also been praised as a blessed tree and fruit in the Holy Quran (Quran, Chapter 24 Al-Noor, Verse 35).

Olives are not used as a natural fruit because of their extremely bitter taste but are rather consumed either as olive oil or table olives [14]. Olive oil market is very significant in the olive industry as approximately 90% of annually produced olives go for oil processing [15]. Table olives and olive oil are very much important in industrial sectors of Palestine, Israel, Spain, and other Mediterranean areas. Spain is the largest producer of olives followed by Italy and Greece. These three countries are producing 60% of the world's total olive production. Outside these, United States and Argentina are the major producers of olive [16, 17].

The therapeutic utilities of* O. europaea* have been indicated in traditional medicine. It has been known to reduce blood sugar, cholesterol, and uric acid. It has also been used to treat diabetes, hypertension, inflammation, diarrhea, respiratory and urinary tract infections, stomach and intestinal diseases, asthma, hemorrhoids, rheumatism, laxative, mouth cleanser, and as a vasodilator. Many phenolic compounds, especially secoiridoids and iridoids [18], and their pharmacological activities have been the focus of attraction for scientists in the last decade [13, 19]. Oleuropein, a major constituent of* O. europaea*, has got much attention and a lot of work has been done on its pharmacological properties [20, 21]. Olive has widely been explored as a functional food [22, 23] with various biophenols [24, 25] and other bioactive constituents [26]. Volatile constituents from olive oil and their applications in flavor development have also been a hot area of the current research [27]. A few reviews have been done on the phytochemistry of olive and olive oil in the last decade [13, 18, 27, 28] but the title plant is under immense investigation by scientists all around the world [28, 29]. Continuous research is in progress to validate its traditional medicinal uses, which is described in detail in the present review.

### 1.1. Botanical Description

**Figure 1:**
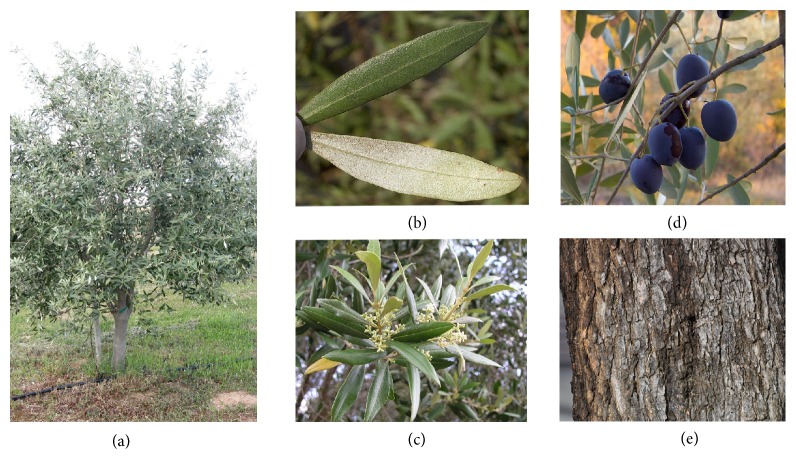
*Olea europaea*: (a) tree; (b) leaves; (c) inflorescence; (d) ripe fruits; (e) stem bark.

The olive tree (Figure 1(a)) is short and thick, generally from trees or shrubs to 10 m in height. Its trunk possesses a large diameter typically bent and twisted. It has many reedy branches with opposite branchlets. The leaves are shortly stalked, lanceolate, sometimes ovate, narrow, oblong, coriaceous, leathery, glabrous, attenuate, apex cute to acuminate, margin entire, pale green above with few scales, and silvery-whitish below in color, petiole 5 mm, and 4–10 cm in length and 1–3 cm wide with 5–11 primary veins on each side of the midrib and raised adaxially (Figure 1(b)) [30]. The flowers are numerous, bisexual or functionally unisexual, small, subsessile, creamy white, and feathery bulled, mostly on the wood of previous year (Figure 1(c)). Calyx is truncate with four little teeth and corolla short with four lobes and is 1-2 mm long. The olive fruit is small of which an outer fleshy part or skin surrounds a shell of hardened kernel. The fruit is ovoid, blackish-violet when ripe, normally 1–2.5 cm long, and small in wild plants than in orchard cultivates (Figure 1(d)) [31]. The bark is pale grey in color (Figure 1(e)) [11].

*O. europaea* is a monoecious plant [32] having both perfect and imperfect flowers [15], so it depends on the wind in which self- or cross-pollination occurs. Cultivated plants are normally pollinated by neighboring cultivars or even the wild form, that is,* O. sylvestris* [33]. Genetically,* O. europaea* is a diploid (2*n* = 46) species [34].

### 1.2. Cultivation

The olive tree is one of the oldest cultivated trees on the planet earth [35]. The cultivation of olive started in ancient times and it dates back more than 7000 years. According to archaeological reports olives were cultivated for commercial purposes in Crete in 3000 BC by the Minoan civilization. The use of olive oil for body health can be found in ancient Greek literature [36]. Earliest olive oil production dates back to some 6500 years ago off the Carmel coast south of Haifa, Israel [37]. Olive trees spread to the West from the Mediterranean area into Italy, Portugal, Spain, Greece, and France [38]. Spanish Conquistadors, in 1560, carried the cuttings and seeds of* O. europaea* to Peru. From there, olive trees spread to Mexico. The Franciscan padres then carried olives and other fruits from San Blas, Mexico, into California. Olive oil production began in San Diego in the next decade [39]. It has been assumed that the cultivars around the Mediterranean originated from wild Mediterranean olive and then spread all around it [40].

Even though olive is now being cultivated in different parts of the world, the Mediterranean region is still serving as the major olive and its oil production area accounting for about 98% of the world's olive cultivation [41]. Nowadays, there are more than 2000 cultivars in the Mediterranean basin which exhibit enormous diversity based on pit size morphology and fruit morphology [42, 43].

## 2. Phytochemistry

**Figure 2:**
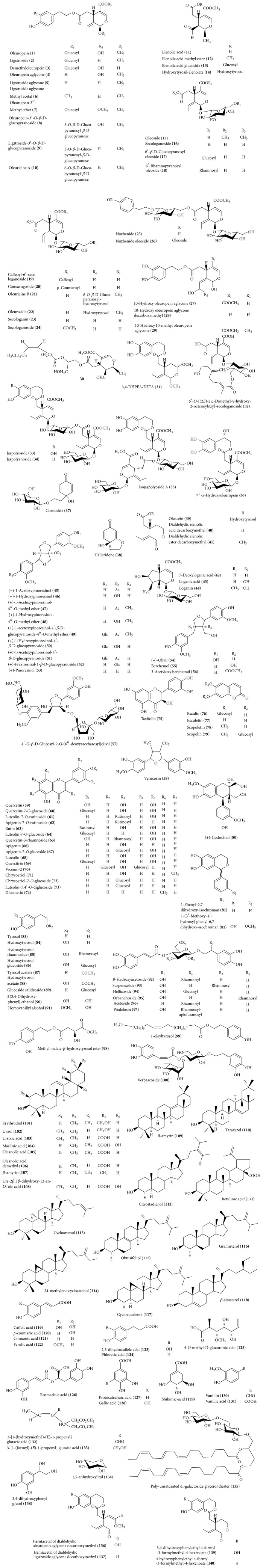
Chemical structures of the compounds isolated from* O. europaea*.

Phytochemical research carried out on* O. europaea* had led to the isolation of flavonoids, flavone glycosides, flavanones, iridoids, iridane glycosides, secoiridoids, secoiridoid glycosides, triterpenes, biophenols [44], benzoic acid derivatives, xylitol, sterols, isochromans, sugars, and a few other types of secondary metabolites from its different parts (Figure 2). Phenolic compounds, flavonoids, secoiridoids, and secoiridoid glycosides [45] are present in almost all the parts of* O. europaea*.

### 2.1. Constituents of the Bark

Lignans like (−)-olivil (**54**), (+)-cycloolivil (**80**), (+)-1-acetoxypinoresinol (**45**), (+)-1-hydroxypinoresinol (**46**), (+)-1-acetoxypinoresinol-4′′-*O*-methyl ether (**47**), and (+)-1-hydroxypinoresinol-4′′-*O*-methyl ether (**48**) have been isolated from the ether extract of the bark of* O. europaea* [46, 47]. Three lignan glucosides, that is, (+)-l-acetoxypinoresinol-4′′-methyl ether-4′-*β*-D-glucopyranoside (**49**), (+)-l-hydroxypinoresinol-4′-*β*-D-glucopyranoside (**50**), (+)-l-acetoxypinoresinol-4′-*β*-D-glucopyranoside (**51**) as well as esculin (**76**), and oleuropein (**1**) were isolated from the bark of* O. europaea* and* O. europaea* ssp.* africana* [48–50]. The bark of* O. europaea* contains (+)-fraxiresinol-1-*β*-D-glucopyranoside (**52**), (+)-l-acetoxypinoresinol-4′-*β*-D-glucopyranoside (**51**) [51], the coumarins esculetin (**77**), scopoletin (**78**), and scopolin (**79**) [48, 49].

### 2.2. Constituents of the Fruits and Seeds

*O. europaea* fruits contain a considerable amount of flavonoids, secoiridoids, secoiridoid glycosides [52], and phenolics such as tyrosol (**83**), hydroxytyrosol (**84**), and their derivatives [53–55]. Some of hydroxytyrosol derivatives like hydroxytyrosol rhamnoside (**85**) [56], hydroxytyrosol glucoside (**86**) [45, 57], and methyl malate-*β*-hydroxytyrosol ester (**98**) [58] were isolated from olive fruit for the first time. Isolation of new tyrosol derivatives, tyrosol glucoside salidroside (**89**), and 1-oleyltyrosol (**99**) [59, 60], along with cornoside (**37**) [20, 52], 2(3,4-dihydroxy-phenyl)ethanol (**90**) [61], halleridone (**38**) [52], and hydroxytyrosol-elenolate (**14**) [54, 61–63] has also been reported from the fruits of* O. europaea*. An important and major phenolic compound present in natural table olives is 3,4-dihydroxyphenylglycol (DHPG) (**138**) which is reported from natural as well as different cultivated olive samples [64, 65].

Galactolipids, triacylglycerols, and fatty acids were isolated from the fruits of* O. europaea* [66–68]. Marra and Giordano reported the isolation of a diacylglycerol (**30**) from olive pulp showing an oleic as well as an elenoic acid residue [69].

Secoiridoids constitute a major portion of the leaves and fruits of* O. europaea*. Oleuropein (**1**) is the most abundantly found secoiridoid glycoside in the fruits of* O. europaea*. A lot of work has been done on isolation, characterization, synthesis, and* in silico* studies of oleuropein [20, 70–72]. Oleuropein may also be used for chemical standardization of the plant and its extracts of medicinal interest [73]. Aouidi et al. developed a method for rapid quantitative determination of oleuropein in* O. europaea* leaves using mid-infrared spectroscopy combined with chemometric analyses [74]. A study of the distribution of phenolic compounds in different parts of the olive fruit showed the presence of oleuropein (**1**), demethyloleuropein (**3**) [75], and verbascoside (**100**) [76] in all parts of the fruit whereas nüzhenide (**25**) [60, 76] was found to be the most concentrated phenolic compound in the seeds of olives [76, 77]. The seeds contain nüzhenide-oleoside (**26**) [60]. Secoiridoids and their glycosides are found in olive fruits and seeds. 3,4-Dihydroxyphenylethyl-[(2,6-dimethoxy-3-ethylidene)tetrahydropyran-4-yl]acetate (**31**) has been reported from the olive fruit [78, 79]. Oleoside (**15**) was isolated from the seeds of olive fruit [60]. Elenolic acid glucoside (**13**) was isolated from olive pulp and was characterized on the basis of LC-MS [53, 57]. 6′-*β*-Glucopyranosyl oleoside (**17**), 6′-rhamnopyranosyl oleoside (**18**), and ligstroside (**2**) were identified in peel, pulp, and fruits of* O. europaea* by electrospray ionization mass spectrometry [57]. Ligstroside aglycone methyl acetal (**6**) was reported from the olives of Hojiblanca cultivar [58].

A large number of flavonoids such as quercetin (**59**) [80], quercetin-7-*O*-glucoside (**60)**, luteolin-7-*O*-rutinoside (**61**) [56, 80], apigenin-7-*O*-rutinoside (**62**) [80, 81], rutin (**63**) [57, 80, 81], vicenin-2 (**70**), chrysoeriol (**71**), chrysoeriol-7-*O*-glucoside (**72**) [80], luteolin-7-*O*-glucoside (**64**) [57, 80], quercetin-3-rhamnoside (**65**), apigenin (**66**) [57], and quercitrin (**69**) [45] have been reported from the fruits and pulp of the olives.

Triterpene alcohols *δ*-amyrin (**109**), taraxerol (**110**), cycloartenol (**113**), and 24-methylene cycloartenol (**114**) along with 4-monomethylsterols, obtusifoliol (**115**), gramisterol (**116**), cycloeucalenol (**117**), and citrostadienol (**112**) were identified in ripening olive fruits [82]. Two secoiridoids with caffeoyl moieties, that is, caffeoyl-6′-secologanoside (**19**) and comselogoside (**20**), were isolated by ultrasound-assisted solid liquid extraction (USLE) of the olive fruits [45]. Different acids include caffeic acid (**119**) [56, 57], 2,3-dihydrocaffeic acid (**123**), phloretic acid (**124**) [55], 4-*O*-methyl-D-glucuronic acid (**125**), and betulinic acid (**111**) [83]. 3-[1-(Hydroxymethyl)-(*E*)-1-propenyl]glutaric acid (**132**) and 3-[1-(formyl)-(*E*)-1-propenyl]glutaric acid (**133**) were isolated from the ethyl acetate extract of olives [84]. Pentacyclic triterpenoids including erythrodiol (**101**), uvaol (**102**), [85], ursolic acid (**103**) [83], and maslinic acid (**104**) [83, 86, 87] were isolated from the fruits of different varieties of* O. europaea*.

Golubev et al. analyzed the tocopherols profile of olive fruits and found that *α*-, *β*-, and *γ*-tocopherols were present as 39.3 mg, 12.2 mg, and 24.0 mg per kilogram of the sample, respectively [88]. Qualitative and quantitative profiling of sugars in vegetal tissues from olive fruits, leaves, and stems resulted in monosaccharides, disaccharides, trisaccharides, sugar carboxylic acids and alcohols, cyclic polyols, and derived compounds [89]. Olive pomace extracts were analyzed by tandem LC-MS and a number of phenolic compounds including 7-deoxyloganic acid (**42**), loganic acid (**43**), loganin (**44**), secologanin (**23**), secologanoside (**24**), taxifolin (**75**), rosmarinic acid (**126**), ferulic acid (**122**), cinnamic acid (**121**), shikimic acid (**129**), protocatechuic acid (**127**), and gallic acid (**128**) were identified [56].

### 2.3. Constituents of the Leaves

Oleuropein has been reported from the methanolic extract of boron deficient leaves, peel, pulp, seeds, and wood of* O. europaea* [20, 47, 75, 90–93]. Oleuropein and other secoiridoids such as secologanoside (**16**), oleoside (**15**), 6′-*E*-*p*-coumaroyl-secologanoside (comselogoside) (**20**), and 6′-*O*-[(2*E*)-2,6-dimethyl-8-hydroxy-2-octenoyloxy]-secologanoside (**32**) have been isolated from the methanolic extract of boron deficient leaves [94]. Oleoside (**15**) has also been isolated from water extract of* O. europaea* leaves [56, 94]. Oleoside (**15**), reported from fruits, is also present in leaves [60]. hydroxytyrosol-elenolate (**14**), elenolic acid methyl ester (**12**), and ligstroside (**2**) were isolated from the chloroform soluble fraction of* O. europaea* leaves [95]. The ethyl acetate extract of olive leaves yielded two new secoiridoid glycosides oleuricine A (**10**) and oleuricine B (**21**) [87]. Oleuroside (**22**) and 3,4-DHPEA-EDA (oleacein) (**39**) were isolated from the aqueous extract of the leaves of* O. europaea* [57, 91, 96].

Isolation from ethyl acetate soluble fraction of* O. europaea* leaves yielded different triterpenoids like *β*-amyrin (**107**) [87, 97], *β*-sitosterol (**118**) [97], oleanolic acid (**105**) [98–103], erythrodiol (**101**) [97, 101, 104], and urs-2*β*,3*β*-dihydroxy-12-en-28-oic acid (**108**). Other triterpene acids isolated from* O. europaea* leaves include betulinic acid (**111**), uvaol (**102**), ursolic acid (**103**), and maslinic acid (**104**) [85].

Flavonoids also constitute some portion of* O. europaea* leaves. Apigenin-7-*O*-rutinoside (**62**), rutin (**63**), and luteolin-7-*O*-glucoside (**64**) were isolated from the leaves of* O. europaea* [80, 81]. Analysis and quantification of leaves of* O. europaea* from different cultivators showed the presence of flavone glycosides, that is, luteolin-7,4′-*O*-diglucoside (**73**), diosmetin (**74**), and apigenin-7-*O*-glucoside (**67**) [81, 91].

A lignan, 4′-*O*-*β*-D-glucosyl-9-*O*-(6′′-deoxysaccharosyl)olivil (**57**), was reported from the leaves of* O. europaea* [105]. A compound never found previously in the vegetal kingdom, that is, 1,5-anhydroxylitol (**134**), was isolated from* O. europaea* leaves [106]. Two new compounds 3,4-dihydroxyphenylethanol-elenolic acid dialdehyde (3,4-DHPEA-EDA) (**39**) and hydroxytyrosol-elenolate (**14**) which are the hydrolysis products of oleuropein (**1**) were isolated from the leaves of* O. europaea* [107]. Besides, hydrocarbons, esters, waxes, triglycerides, tocopherols, esterols, lineal, terpenic alcohols, and terpenic dialcohols have also been reported from the hexane extract of* O. europaea* leaves [108].

### 2.4. Constituents of Olive Oil and EVOO

Olive oil is a very rich source of biophenolic compounds and possesses many interesting biological properties [92, 109]. Presence of hydroxytyrosol (**84**), hydroxytyrosol acetate (**88**) [110, 111], and 3,4-dihydroxyphenylethyl-[(2,6-dimethoxy-3-ethylidene)tetrahydropyran-4-yl]acetate (**31**) has been reported in EVOO (extra virgin olive oil) and olive oil [56, 111]. LC-NMR analysis of alperujo, fresh solid waste of two-phase olive oil extraction system, showed the presence of *β*-hydroxyacetamide (**92**), suspensaside (**93**), hellicoside (**94**), orbanchoside (**95**), acetoside (**96**), and wedelosin (**97**) [112]. Hydroxytyrosol-elenolate (**14**), oleuropein aglycone (**4**), ligstroside aglycone (**5**), 10-hydroxy oleuropein aglycone (**27**), 10-hydroxy oleuropein aglycone decarboxymethyl (**28**), 10-hydroxy-10-methyl oleuropein aglycone (**29**), elenolic acid (**11**), dialdehydic elenolic acid decarboxymethyl (**40**), dialdehydic elenolic ester decarboxymethyl (**41**), vanillic acid (**131**),* p*-coumaric acid (**120**), and (+)-pinoresinol (**53**) have been reported from olive oil and EVOO obtained from various cultivators [56, 57, 95, 111, 113, 114]. Christophoridou et al. identified various phenolic compounds in olive oil by coupling HPLC with post column solid-phase extraction to nuclear magnetic resonance spectroscopy (LC-SPE-NMR). These include tyrosol acetate (**87**), vanillin (**130**), homovanillyl alcohol (**91**), (+)-1-acetoxypinoresinol (**45**), berchemol (**55**), 3-acetyloxy berchemol (**56**), hemiacetal of dialdehydic oleuropein aglycone decarboxymethyl (**136**), hemiacetal of dialdehydic ligstroside aglycone decarboxymethyl (**137**), and verucosin (**58**) [110]. Flavonoids such as apigenin (**66**) and luteolin (**68**) [80, 81], other phenolics including 3,4-dihydroxyphenylethyl-4-formyl-3-formylmethyl-4-hexenoate (**139**) and 4-hydroxyphenylethyl-4-formyl-3-formylmethyl-4-hexenoate (**140**) [75, 111], and isochromans, that is, 1-phenyl-6,7-dihydroxy-isochroman (**81**) and 1-(3′-methoxy-4′-hydroxy)phenyl-6,7-dihydroxy-isochroman (**82**) [115], have also been reported from olive leaves, oil, and EVOO. A di-galactoside of a poly-unsaturated diester of the glycerol (**135**) was also isolated from olive oil [67].

### 2.5. Constituents of the Wood

Many important secondary metabolites have been reported from the wood of* O. europaea*. Ligstroside (**2**), a major component of olive fruit and seeds, is also present in the wood [76]. Pérez-Bonilla et al. isolated oleuropein-3′′-methyl ether (**7**), 7′′-*S*-hydroxyoleuropein (**36**), oleuropein-3′-*O*-*β*-D-glucopyranoside (**8**), ligstroside-3′-*O*-*β*-D-glucopyranoside (**9**), jaspolyoside (**33**), jaspolyanoside (**34**), and isojaspolyoside A (**35**) from the ethyl acetate fraction of olive wood [116]. An iridoid glycoside, 7-deoxyloganic acid (**42**), was isolated from ethyl acetate extract of olive wood [47]. A new compound known as oleanolic acid demethyl (**106**) has been reported from the stem of* O. europaea* [117].

## 3. Traditional and Contemporary Uses

*O. europaea* has a number of traditional and contemporary uses in medicine. Some of its exemplary uses are given below while the others are summarized in Table 2.* O. europaea* is extensively used in traditional medicine for a wide range of ailments in various countries. Its bark, fruits, leaves, wood, seeds, and oil are used in different forms, alone or sometimes in combination with other herbs. Oil of seeds is taken orally as a laxative and also applied externally as a balm for inflammation [118]. Decoctions of dried leaves and fruit are used orally to treat diarrhea, respiratory and urinary tract infections, stomach and intestinal diseases, and as mouth cleanser [119]. Continuous application of olive oil is also useful to prevent hair loss [120]. In Canary Islands, the infusion prepared from olive leaves is taken orally as a hypotensive while it is administered through rectum for hemorrhoids [121, 122]. In East-Africa the infusion of the bark of olive tree is taken for tapeworm infestation after soaking for whole night [123]. In Greece hot water extract of olive leaves is taken orally to treat high blood pressure [124]. In Italy, the extract of the essential oil of fruit is taken orally to treat renal lithiasis. It is applied externally to treat burns, ores, and rheumatism and to promote circulation [125]. Infusion of fresh leaves is also taken as anti-inflammatory [126]. Tincture of olive leaves is taken in Italy as a febrifuge [127] and applied externally as an emolument for ingrown nails and a restorer of epithelium [128]. In Japan olive leaves are taken orally for stomach and intestinal diseases and their essential oil is used orally for constipation and liver pain [119].

Olive oil has been taken orally to treat hypertension and agitation, as a laxative and vermicide in the United States [129]. Boiled extract of fresh or dried leaves is taken orally to treat asthma [124] and hypertension and to induce diuresis [130, 131]. The oil is applied externally over fractured limbs [132] and fruit is also known to be a skin cleanser [133]. Infusion of leaves is taken orally to reduce fever [127] and as anti-inflammatory tonic [126].* O. europaea* leaf preparations are used as a common remedy for gout in Mediterranean folk medicine [134]. Leaves of* O. europaea* are used in Tunisian folk medicine as a remedy for many inflammation types and bacterial infections such as gingivitis, otitis, icterus, and cough [135]. Fruits and leaves of* O. europaea* are used to treat hemorrhoids and rheumatism, and as vasodilator in vascular disorders [136]. Infusions of leaves are used as ointment to treat eye infections or as mouthwash to relieve sore throat [137]. Hot water extract of the fresh leaves of* O. europaea* is taken orally to treat hypertension and to induce diuresis in Brazil [131]. Decoction of leaves is used in Morocco to treat hypertension and diabetes [138]. Olive oil is mixed with lemon juice and is used to treat gallstones [139]. Decoction or infusion of the fruits and leaves is used in Palestine to treat diabetes [140, 141]. In Algeria the fruits and leaves of* O. europaea* are used to cure diabetes and hypertension [142].

## 4. Pharmacology

### 4.1. Antidiabetic Activities

The ethnomedical use of* O. europaea* in treatment of diabetes has been validated in several experimental studies (summarized in Table 3). The plant has been used by the folk medical practitioners to treat diabetes. Al-Azzawie and Alhamdani proposed that antidiabetic patients may be treated with good antioxidants as the relief in oxidative stress reduces the blood glucose levels. So they treated hypoglycemic alloxan-diabetic rabbits with oleuropein, a powerful antioxidant agent present abundantly in olive leaves and fruit, to reduce their oxidative stress. The diabetic rabbits were treated with oleuropein (20 mg/Kg body weight) for up to 16 weeks. After treatment it was observed that the blood glucose levels along with most of the antioxidants were restored to the values near to the normal control rabbits. The study proved the effects of oleuropein as antihyperglycemic and antioxidative agent [143].

It was believed that the antidiabetic effects of* O. europaea* are solely due to the major secoiridoid glycoside, oleuropein (**1**), present in its different parts. Sato et al. isolated the named oleanolic acid (**105**) and showed that it was an agonist for TGR5 (the first cell surface receptor activated by bile acids), a member of G-protein coupled receptor, and it significantly lowered serum glucose and insulin levels in mice fed with a high fat diet and also enhanced glucose tolerance. They suggested on the basis of their findings that both oleuropein (**1**) and oleanolic acid (**105**) were responsible for the antidiabetic effects of olive leaves [144]. Later on the findings of Al-Azzawie and Alhamdani were supported by the studies of antidiabetic and antioxidant effects of oleuropein (**1**) and hydroxytyrosol (**84**), isolated from the leaves of* O. europaea*, in alloxan-induced diabetic rats. The rats suffered from hyperglycemia, increased lipid peroxidation, hypercholesterolemia, and depletion in the antioxidant enzymes activities. After administration of oleuropein and hydroxytyrosol rich extracts having approximately 8–16 mg/Kg body weight of each compound for four weeks, the serum cholesterol and glucose levels of the diabetic rats were significantly lowered and antioxidant enzymatic activities were restored. It was suggested that the antidiabetic effect of oleuropein and hydroxytyrosol might be due to their ability to restrain the oxidative stress which is widely associated with pathological complications of diabetes [145].

A comparative study was conducted to check the effect of olive leaves extract in normal and diabetic rats. The diabetes was induced in rats by injecting streptozotocin. The rats were orally administered with olive leaves extracts at different doses of 100, 250, and 500 mg/Kg of body weight, while the reference drug, Glibenclamide, was given at a dose of 600 *μ*g/Kg for 14 days. The antidiabetic effect of the extract was more potent than the reference drug, Glibenclamide. More interestingly, the extracts, besides lowering the serum glucose, total cholesterol, urea, uric acid, triglycerides, and creatinine, also increased the serum insulin levels in diabetic and not in normal rats. The study proposed that olive leaf extract may be used as an antidiabetic agent [146]. These studies demonstrated the effect of olive leaves extract and oleuropein on diabetes but did not explain the cellular mechanisms of these effects which are responsible for controlling blood glucose levels, including the effects on insulin secretion and any relation with cellular redox state. Cumaoğlu et al. investigated the effects of biophenolic olive leaf extract (OLE) and oleuropein (**1**) on intracellular oxidative stress, apoptosis, necrosis, and insulin secreting function in the INS-1 cell line. The cells were exposed to H_2_O_2_ for 45 minutes which resulted in a depressed glucose-stimulated insulin secretion, increased apoptotic and necrotic cell death, inhibited glutathione peroxidase activity (GPx), and stimulated the catalase activity that were associated with increased intracellular generation of reactive oxygen species (ROS). These cells were then treated with OLE and the reference, oleuropein. Both improved partly necrotic and apoptotic cell death and inhibited the ROS generation and improved the secretion of insulin in H_2_O_2_-exposed cells. The results showed that oleuropein had more pronounced effect on insulin secretion than OLE [147]. It was probably due to the presence of other biophenols in OLE which suppressed the properties of oleuropein which it might express alone [148].

A new compound has been reported from the stem of* O. europaea* which is an isomer of oleanolic acid (**105**) and was known as oleanolic acid demethyl (**106**). This compound exhibited excellent inhibitory activities* in vitro* against *α*-amylase and lipase enzymes [117]. Recently, the effect of oleanolic acid on streptozotocin-induced diabetic neuropathy in Sprague Dawley rats has been studied [149]. The rats were divided into control group and diabetic group and were daily treated with 20, 40, and 60 mg/Kg doses of oleanolic acid up to four weeks. The results indicated that 60 mg/Kg dose of oleanolic acid was more active which reduced the oxidative stress in the kidneys and inhibited the neuropathy induced alterations. Oleanolic acid proved to act* via* multiple mechanisms, that is, antioxidant mechanism,* via* inhibition of generation of advanced glycation end products, improving the insulin secretion. Al-Qarawi et al. studied the effect of freeze-dried extract of olive leaves on the pituitary-thyroid axis in rats and showed that the thyroid hormones in turn decrease the levels of cholesterol and sugar [150].

A placebo-controlled crossover trial was conducted in New Zealand to evaluate the effect of supplementation with olive leaf biophenols on insulin action and cardiovascular risk factors in middle-aged overweight men. 46 participants were selected to receive capsules of olive leaves extract or placebo for 12 weeks. The results concluded that OLE (olive leaf extract) supplementation resulted in a 15% improvement in insulin sensitivity compared to placebo. There were, however, no effects on lipid profile, ambulatory blood pressure, body composition, or liver function [151].

### 4.2. Anticancer Activities

Constituents of* O. europaea* have shown very good anticancer activities on various types of cancers [152]. The anticancer activities of* O. europaea* are summarized in Table 4. Juan et al. investigated the antiproliferative and apoptotic activities of erythrodiol (**101**) in human colorectal carcinoma HT-29 cells [153]. It inhibited the cell growth without any toxicity in a concentration range of 100 *μ*M in colon adenocarcinoma cells. Similarly, studies have been conducted on water and methanolic extracts of olive leaves against cancer and endothelial cells. These crude extracts were found to inhibit cell proliferation of human breast adenocarcinoma (MCF-7), human urinary bladder carcinoma (T-24), and bovine brain capillary endothelial (BBCE). Upon phytochemical analysis of these extracts oleuropein was found to be the most abundant compound along with other phenolics and flavonoids. Then the isolated pure compounds luteolin 7-*O*-glucoside (**64**), oleuropein (**1**), hydroxytyrosol (**84**), and hydroxytyrosol acetate (**88**) were also subjected to cell proliferation assays. It was concluded that in pure form, these compounds were active in low concentrations [154].

Allouche et al. investigated effects of erythrodiol (**101**), uvaol (**102**), oleanolic acid (**105**), and maslinic acid (**104**) on cytotoxicity, apoptosis, cell proliferation, cell cycle, reactive oxygen species (ROS), and oxidative DNA damage on human MCF-7 breast cancer cell line. The results showed that erythrodiol, uvaol, and oleanolic acid have a significant cytotoxic effect and inhibit proliferation in a dose- and time-dependent manner. All these compounds protected against oxidative DNA damage at the concentration 10 *μ*M. Overall results showed that the stated triterpenes have significant natural defense against human breast cancer [155]. The ethanolic extract of the leaves of a Lebanese olive plant were tested on human leukemic cell line. Cytotoxicity of the various concentrations of this extract was also determined. WST-1 proliferation kit and [^3^H]-thymidine incorporation method was used for antiproliferation assay. The extract showed a concentration-dependent activity by inducing apoptosis. Antioxidant activity of the extract was also determined by DPPH scavenging method [156]. Antitumor effects of oleanolic acid (**105**) have been investigated recently both* in vitro* and* in vivo*. HepG2 cells were injected subcutaneously to mice to establish transplanted tumors. Oleanolic acid exhibited inhibitory effect on hepatocellular carcinoma through induction of apoptosis and cell cycle arrest both in transplanted tumors and in HepG2 cells. The results demonstrated that oleanolic acid has significant antitumor activities in hepatocellular carcinoma both* in vitro* and* in vivo* models [157]. Recently, Burattini et al. investigated the effects of hydroxytyrosol and its ester hydroxytyrosol laurate in U937 cells, a human monocytoid cell line, and in C2C12 myoblasts (a murine proliferating muscle cell model) after apoptotic death induction. H_2_O_2_ at known concentrations was used to induce apoptosis. The results revealed that hydroxytyrosol laurate has a greater protective antioxidant effect against H_2_O_2_ treatment than hydroxytyrosol [158]. The effect of olive leaf biophenols like oleuropein (OLP) and hydroxytyrosol (HT) was studied on tumor growth in breast cancer. It was observed that the treatment with 150 and 225 mg/Kg/day of OLE significantly reduced the tumor volume and weight [159].

Maslinic acid (**104**), a triterpenoid reported from olive leaves and fruit, is an antitumor agent which showed potent antiproliferative properties against the HT29 colon cancer cells [160]. It affects the levels of structural proteins necessary to the maintenance of cell structure and function and inhibits the cell growth by G1 cell cycle arrest, indicating its therapeutic potential in colon cancer therapy [161, 162].

Oleuropein (**1**), the most prominent phenolic compound present in the olive tree, has been evaluated for its activity on human colon adenocarcinoma (HT-29) cells in comparison with its hydrolysis product hydroxytyrosol (**84**) to elucidate the underlying mechanisms of action. The sulforhodamine B (SRB) assay was used to detect cell proliferation while flow cytometry and Western blot procedures were used in order to assess apoptosis and changes in regulation of HIF-1*α* and p53, respectively. Cell growth inhibition results showed hydroxytyrosol more active than oleuropein but in case of apoptotic population and oleuropein showed a significant increase. The results demonstrated that oleuropein limits the growth and induces apoptosis in HT-29 cells* via* p53 pathway activation adapting the HIF-1*α* response to hypoxia [163]. Oleuropein (**1**) and OLE were studied for their effects on unstimulated lymphocytes. It was noticed that it did not produce any cytotoxic effects but had a stimulatory effect and induced high proliferation in lymphocytes in a concentration dependent manner [164].

### 4.3. Antimicrobial Activities

*O. europaea* has been used as a folk remedy for the cure of numerous infectious disorders of bacterial, fungal, and viral origin. Several studies have been carried out in the past validating the antimicrobial and antiviral potential of* O. europaea* (summarized in Table 5) [165]. Kubo et al. characterized a series of long-chain *α*,*β*-unsaturated aldehydes from* O. europaea* fruit and its oil flavor for their antimicrobial activities and found them active against a broad spectrum of microbes [166]. Pereira et al. tested the extracts of various table olives from different cultivators of Portugal for their antimicrobial potential and found that besides having good antioxidant potential, olive phenolics possess good antimicrobial activity suggesting that these table olives may be good candidates against bacteria responsible for human gastrointestinal and respiratory tract infections [167].

It was observed that olive brines have antimicrobial activity against lactic acid fermentation. A study was designed to investigate their antimicrobial activity. The samples of these olive brines were analyzed by HPLC, electrospray ionization mass spectrometry, and NMR spectroscopic studies. Among the identified compounds, the dialdehydic form of decarboxymethyl elenolic acid linked to hydroxytyrosol (**39**) showed the strongest antilactic acid bacteria activity and its presence in olive brines also explained the growth inhibition of these bacteria during fermentation of olives [168]. Ethanolic extract of the fresh leaves of* O. europaea* was tested against* Artemia salina* and was found to be active. Its lethal dose was 164 pg/mL [169]. Sudjana et al. investigated the commercial olive leaf extract against a wide range of microbes (*n* = 122) using agar dilution and broth microdilution techniques. The results showed that olive leaf extract was only active against* Campylobacter jejuni*,* Helicobacter pylori*, and* Staphylococcus aureus* showing that it is not a broad spectrum antimicrobial agent [170]. Olive leaves' phenolic compounds were also evaluated against several microorganisms that are causative agents of human intestinal and respiratory tract infections including Gram positive bacteria (*Bacillus cereus*,* B. subtilis*, and* Staphylococcus aureus*), Gram negative bacteria (*Pseudomonas aeruginosa*,* Escherichia coli*, and* Klebsiella pneumoniae*), and fungi including* Candida albicans* and* Cryptococcus neoformans*. The extracts showed an unusual combined antibacterial and antifungal activity at low concentrations against the stated species which suggest their potential as nutraceuticals [167]. The olive leaf extracts exhibited relatively high antimicrobial activity against* Salmonella typhimurium*,* Escherichia coli*,* Staphylococcus aureus*,* Bacillus cereus*,* Listeria monocytogenes*, and* Pseudomonas aeruginosa* using disc diffusion method [171]. The* in vitro* antimicrobial activity of aqueous, acetone, diethyl ether, and ethyl alcohol extracts of olive leaves was investigated against a number of microorganisms. The aqueous extract of olive leaves had no antibacterial effect against the test microorganisms, while acetone extract inhibited several microorganisms including* Salmonella enteritidis*,* Klebsiella pneumoniae*,* Bacillus cereus*,* Escherichia coli*,* Streptococcus thermophiles*,* Enterococcus faecalis*, and* Lactobacillus bulgaricus* [172]. O.-H. Lee and B.-Y. Lee studied the combined effect of olive leaf phenolics and the isolated compounds against several microbial strains. The results indicated the active components as oleuropein (**1**) and caffeic acid (**119**) and it was concluded that the combined olive phenolics have a significantly higher antimicrobial effect than the isolated phenolics [173]. Other studies have been conducted to observe the antimicrobial activities of olive leaves' extracts. Oleuropein was found the most effective compound while syringic acid was found inactive. Aqueous extract was inactive against* Bacillus cereus* while acetone extract was found active [172, 174]. Volatile fractions from fresh and dried leaves of three Tunisian olive cultivators were subjected to GC-MS analysis and their antibacterial and antifungal activities were evaluated. All the fractions showed significant antibacterial and antifungal activities, although there were differences in the responses of different cultivators to the microorganisms because of variability of the composition [175]. Another study was designed to investigate the effects of alcoholic extract of olives on the bacterial communication system, expressed as quorum sensing activity. The extract was tested for the stated activity by* Chromobacterium violaceum* assay using the agar cup diffusion method. The extract of olives was not found active for antiquorum sensing activity [176].

The bactericidal and fungicidal activities of salt-free table olive solutions were evaluated against several phytopathogenic microorganisms [177]. The results demonstrated that the storage solutions of black ripe olives showed evident antibacterial activity against species of* Erwinia*,* Clavibacter*,* Agrobacterium*, and* Pseudomonas* while washing waters from Spanish-style green olives were not so effective. These solutions also showed fungicidal activity against species of* Phytophthora*,* Colletotrichum*,* Alternaria*,* Botrytis*, and* Pestalotiopsis*. These activities were related to the glutaraldehyde like compounds such as the dialdehydic form of decarboxymethyl elenolic acid in these solutions. These findings suggested that olive waste waters may be used in agriculture for pests management [178]. Antifungal activities of aqueous, acetone, ethyl acetate, and methanolic extracts of olive leaves were investigated on 30 different fungal strains using disc diffusion method. The aqueous extract showed the highest activity and was active against 10 fungal strains. When sensitivity was compared,* Alternaria parasiticus* was the most resistant strain while* Alternaria wentii* was the most sensitive one [179]. A study was conducted to evaluate the antifungal activity of some aliphatic aldehydes [hexanal, nonanal, (*E*)-2-hexenal, (*E*)-2-heptenal, (*E*)-2-octenal, (*E*)-2-nonenal] from olive fruit against various strains of* Trichophyton mentagrophytes*,* Microsporum canis*, and* Candida* spp. These compounds were also tested for their ability to inhibit elastase, a virulence factor essential for the dermatophytes' colonization. The aldehydes showed a broad spectrum activity and inhibited all the tested strains except* Candida* spp. (*E*)-2-octenal and (*E*)-2-nonenal inhibited the elastase activity in a concentration dependent fashion [180]. A similar type of study has shown that volatiles from Tunisian olive cultivars (Chemlali and Neb Jmel) were found active against a number of bacteria and fungi [181]. Maslinic acid (**104**), a pentacyclic triterpenoid obtained from the pressed fruits of olives, has been studied against tachyzoites of* Toxoplasma gondii*. The parasite when treated with maslinic acid showed inhibition of some of the proteases in a dose-dependent manner. This protease activity is required by the parasite for cell invasion. Hence, maslinic acid can be a good agent to inhibit this parasite [182]. Micol et al. described the antiviral activity of a commercial olive leaf extract (LExt) and its main constituent oleuropein against the viral hemorrhagic septicemia virus (VHSV). Both of these showed good activity against VHSV before infection as well as in postinfection treatment. Both the extracts were safe on health and environment so these may be used as promising natural antiviral agents [183].

### 4.4. Antioxidant Activities

Reactive oxygen and nitrogen species are essential for energy supply, chemical signaling, detoxification, and immune function and are continuously produced in the human body and their amount is carefully controlled under the action of endogenous enzymes such as superoxide dismutase, glutathione peroxidase, and catalase. With an overproduction of these reactive species, an exposure to external oxidant substances, or a failure in the defense mechanisms, damage to valuable biomolecules (DNA, lipids, proteins) may occur [26]. This damage has been associated with an increased risk of cardiovascular disease, cancer, and other chronic diseases. Hence, antioxidants are required to prevent from oxidative damage and chronic diseases [184]. Antioxidant activities of* O. europaea* have been summarized in Table 5. Le Tutour and Guedon investigated the antioxidant activity of oleuropein, hydroxytyrosol, and tyrosol from the leaves of* O. europaea* in comparison with vitamin E and BHT. Oleuropein and hydroxytyrosol showed high antioxidant activity while tyrosol showed neither antioxidant nor prooxidant activity [185]. Oleuropein was also evaluated for its antioxidant activity* in vitro* using chemiluminescence assay and was found to have remarkable antioxidant activity [186]. Later on Fogliano et al. evaluated the antioxidant activities of two virgin olive oils obtained from same olive batch but processed with different hammer crushing conditions and found that the efficacy of the olive oil which was processed under higher hammer crushing rate was higher than the other [187]. In another study, antioxidant activity of different phenolic compounds isolated from the leaves of* O. europaea* was determined. These compounds included oleuropein, verbascoside, luteolin-7-glucoside, apigenin-7-glucoside, diosmetin-7-glucoside, luteolin, diosmetin, rutin, catechin, tyrosol, hydroxytyrosol, vanillin, vanillic acid, and caffeic acid. Among these, the flavonoids rutin, luteolin, and catechin showed the highest activity against ABTS radicals [188].

Briante et al. proposed a fast method for the biotransformation of the leaves extract of* O. europaea* by a thermophilic *β*-glycosidase enzyme immobilized on chitosan to get extracts with high amounts of hydroxytyrosol. To confirm the transformation, the resulting extracts were evaluated for their antioxidant activities in comparison with the original olive leaves extract. The resulting eluates showed a higher antioxidant activity than their extract of origin due to the presence of a higher concentration of simple biophenols [189]. A similar kind of comparative study was performed between extra virgin olive oils (EVOOs) of different degrees of degradation. It was concluded that the EVOO with a low degradation level of 3′,4′-DHPEA-EDA and a lower content of 3′,4′-DHPEA than the oils with intermediate and advanced degradation levels were 3–5 times more efficient as DPPH scavengers and 2 times more efficient as inhibitors of the XOD-catalyzed reaction than oils with intermediate and advanced degradation levels [190]. Similar studies have also been carried out on EVOO showing that the compound responsible for the antioxidant properties of the EVOO was 3,4-DHPEA-EDA [191]. Ranalli et al. elucidated the antioxidizing potency of phenolics present in olive oil mill waste waters using reference standards of natural and synthetic origin, commonly used as food preservatives. The results showed that many phenolics present in olive oil mill waste water have good antioxidizing capacities and could replace some less safe synthetic antioxidants used to preserve food items industrially [192]. The effect of maslinic acid (**104**), obtained from olive pomace, on the vulnerability of plasma membrane to lipid peroxidation has been investigated both* in vitro* and* in vivo* in rats. It was concluded that maslinic acid may offer some resistance to oxidative stress in animals [193]. LC-MS screening of freeze-dried olives from Italian cultivators was performed along with their antioxidant profile showing differences in antioxidant behavior of olives of different cultivators [194]. In a similar study, the phenolic components of* O. europaea* were identified by HPLC and antioxidant activities of different isolates were measured in sunflower oil by measuring peroxide values [195].

Antioxidant activities of different parts of* O. europaea* have been evaluated in different studies using hydroxyl and 2,2-diphenyl-1-picrylhydrazyl (DPPH) radicals as reference. These include olive leaves infusion [196], ethanolic extract of olive leaves [197, 198], olive fruit and leaves [136, 199], aqueous olive leaf extract [171, 200], and olives from Dhokar olive cultivators [201]. All the extracts exhibited very good antioxidant activities. The phenolic compounds composition and antioxidant activities of table olives from different cultivators of Portugal namely, Trás-os-Montes e Alto Douro, Galega, Negrinha de Freixo, Azeitona de Conserva Negrinha de Freixo olives, and Azeitona de Conserva de Elvas e Campo Maior olives, were examined using trolox as a reference. All the extracts showed very good radical scavenging activities which proved them to be a healthy diet supplement [167, 202]. In a different study extra virgin olive oils of different varieties including arbequina, hojiblanca, and picual were taken and their* n*-hexane/ethanol (20 : 80) extracts were assessed for their radical scavenging activities using DPPH (2,2-diphenyl-1-picrylhydrazyl), ABTS (2,2′-azino-bis (3-ethylbenzthiazoline-6-sulfonic acid)), hydroxyl radical, hydrogen peroxide, and superoxide anion inhibitory activities. The results indicated that EVOOs possess greater antioxidant potential than pure olive oils [203]. Tunisian olives were subjected to antioxidant assays using trolox equivalent antioxidant capacity (TEAC) protocol and were found to have antioxidant capacity between 212 and 462 *μ*M TEAC/g of dry weight [204].

Conde et al. subjected* O. europaea* pruning to liquid hot water treatment (LHW) and steam explosion (SE) and noticed that hot water treatment enhanced the release of phenolic compounds in the aqueous phase. Syringol and syringaldehyde were among the most abundant volatile compounds and oleuropein, hydroxytyrosol, syringaldehyde, and tyrosol were the most abundant phenolics identified by HPLC. The extracts showed a promising antioxidant activity which was hardly affected by the temperature of the treatment [205]. Olive trees in Portugal were sprayed with different copper formulations in order to protect the trees from different fungal diseases. Later on the leaves of those trees were analyzed for their copper content by atomic absorption spectrometry. It was observed that the samples with more copper content have a decreased level of phenolic compounds and in turn less radical scavenging capacity [206].

Four major phenolic compounds present in olive oil, namely, hydroxytyrosol (**84**), oleuropein (**1**), hydroxytyrosol-elenolate (**14**), and 3,4-dihydroxyphenylethanol-elenolic acid dialdehyde (**39**), were studied for their protective effect on RBCs against oxidative damage. All four compounds significantly showed the protective effect for RBCs from oxidative damage in a dose-dependent manner [207]. Abaza et al. conducted a study to test the effect of extraction solvent on antioxidant activity. Antioxidant activities were evaluated using DPPH and ABTS methods. Results revealed that methanol is recommended for the extracts with high level of flavonoids and antioxidant activities [208]. The protective effects of olive leaf extract on genotoxicity and oxidative damage in cultured human blood cells are also studied. The olive leaf extract at all doses did not induce any significant changes in genotoxicity; however, it increased total antioxidant capacity in plasma* in vitro*. The ethanol extract at 100 mg/L dose induced genoprotective activity* via* increase in antioxidant activity, scavenging of free radicals, and inhibition of oxidative stress [209]. The effect of ripening on the antioxidant activities of the olive pericarp was examined in eleven different olive cultivars grown in southern Italy. The geranylgeranyl reductase transcript levels along with the biophenols and tocopherols were studied and it was observed that an inverse relation exists between them. At ripening stages of pericarp, the tocopherol levels increased gradually while biophenolic levels decreased significantly confirming the significant properties of antioxidants in the ripe olive drupes, hence establishing their nutritional value [210]. Antioxidant properties of phenolic compounds of olive fruits pulp from* chamlal* variety were evaluated in comparison with vitamin C. Hydrogen peroxide (H_2_O_2_) scavenging and iron reduction tests were performed to determine the antioxidant activities. All the tested substances exhibited a strong reducing power. The protective effect of the extracts against lipids and proteins peroxidation was also studied. The olive pulp showed a significant activity for the protection of lipids peroxidation [211]. Petridis et al. evaluated the antioxidant activities of fruits and leaves of eleven different olive cultivars and found that significant differences existed between the activities of different cultivars and the oleuropein content and antioxidant activities of leaves were higher than the fruits [212]. The infusions of leaves of* O. europaea* from Mediterranean region were evaluated for their radical scavenging abilities using DPPH radicals in mice and were found good in reducing the oxidants in the body [196]. The extracts of olive leaves have also been found to be good antioxidants against the oxidation of soybean oil under microwave heating. Microwave heating causes severe nutritional and quality losses and increased the peroxide values. Olive leaves extracts made availability of vitamin E in the test samples ensuring protection against formation of peroxides [213]. Machado et al. studied the effect of irrigation and rain water on the growth, total phenolic content, antioxidant activities, and L-phenylalanine ammonia-lyase (PAL) activity of Portuguese olive tree cultivar Cobrançosa. They found that the rain-fed olive trees during the maturation of their fruits yielded in higher biophenolic content and PAL activity than the irrigated olive trees [214]. An experiment was designed to study the effect of dietary olive leaves on lipid and protein oxidation of refrigerated stored n-3 fatty acids-enriched pork. It was observed that the supplementation of meat with olive leaves decreased lipid oxidation but exerted no effect on protein oxidation in both raw and cooked meat [215]. The hydroxyl-isochromans (**81** and** 82**) were investigated for their antioxidant power and antiplatelet activity. The results suggested them as very good antioxidants and inhibitors of platelet aggregation and thromboxane release, so the use of olive oil can reduce the risk of vascular diseases [216].

### 4.5. Enzyme Inhibition Activities

The use of olive in folk medicine to treat the inhibition of enzymes involved in various ailments is old. In Mediterranean folk medicine, the preparation of olive leaf has been used as a common tonic for gout [134]. Flemmig et al. investigated the possible inhibitory effects of 80% ethanolic dry olive leaf extracts and nine isolated compounds from it against xanthine oxidase (XO), an enzyme which is a cause of gout. Allopurinol was used as a reference drug in this study. Olive leaf extract as well as several of its phenolics showed xanthine oxidase inhibitory activity. The flavone apigenin (**66**) showed the strongest XO inhibitory activity while oleuropein (**1**), caffeic acid (**119**), luteolin-7-*O*-*β*-D-glucoside (**64**), and luteolin (**68**) also showed significant contribution in XO inhibition [134]. Water extract of the fresh terminal branches of* O. europaea* ssp.* africana* inhibited peptidase and glycosidase enzyme activities which are produced by the periodontopathic bacteria* Porphyromonas gingivalis*,* Bacteroides intermedius*, and* Treponema denticola* [217]. The aqueous extract of olive leaves was tested for Angiotensin Converting Enzyme (ACE) inhibition activity* in vitro*. Due to significant activity, a bioguided isolation was performed to isolate the main component responsible for the ACE inhibition which was oleacein (**39**) while the other isolates did not show ACE inhibitory activity [96]. A series of *α*,*β*-unsaturated aldehydes, characterized from olive oil flavor, were found to inhibit the enzyme tyrosinase which catalyzes the oxidation of L-3,4-dihydroxy phenylalanine (L-DOPA). These aldehydes were proved to be noncompetitive inhibitors of tyrosinase and showed low toxicity on brine shrimp test [218].

### 4.6. Antihypertensive and Cardioprotective Activities

Hypertension is the cause of heart diseases and it may cause stroke of the arteries, peripheral arterial diseases, and chronic kidney diseases if not treated. Many natural products have been found effective against hypertension. The cardiotonic effects of three triterpenoids, namely, uvaol (**102**), ursolic acid (**103**), and oleanolic acid (**105**) isolated from the leaves of* O. europaea*, were examined. Oleanolic acid and uvaol showed a significant, dose-response vasodepressor effect; therefore, olive oil was suggested as a natural and cheap source of controlling hypertension [219]. A random parallel clinical trial was conducted on the patients of stage-1 hypertension to evaluate the antihypertensive effects of olive leaf extract in comparison with the reference drug Captopril. Olive leaf extract was administered at a dose of 500 mg twice a day while the control group was treated with 25 mg Captopril twice a day until 8 weeks. All the patients showed a significant decrease in their systolic as well as diastolic blood pressure after treatment and the patients treated with olive leaf extract showed better results than Captopril [220]. Ethanolic extract of olive leaf, upon injection intragastrically to mice at a dose of 250 mg/Kg body weight, was found active against 11-deoxycorticosterone acetate induced hypertension [221]. In another study, the ethanolic or chloroform extract of olive leaves showed a slow decrease in blood pressure in moderate hypertension after prolonged use [222]. It was observed that infusion of the dried olive leaves upon intravenous administration to mice remained ineffective against normal blood pressure but had a decreasing effect in rennin and norepinephrine induced hypertension [223]. The triterpenoids isolated from the leaves of* O. europaea* from African, Greece, and Cape Town cultivars were tested against insulin-resistant rats for hypertension at a dose of 60 mg/Kg for six weeks. The results showed that main active constituents were oleanolic acid and ursolic acid which prevented the hypertension and atherosclerosis and also improved the insulin resistance of the test rats [224].

A clinical trial of aqueous extract of olive leaves was carried out on hypertensive patients. Patients were treated with 1.6 *μ*g/day olive leaves extract up to three months after 15 days treatment based on placebo. A significant decrease in their blood pressure was noticed after treatment without any side effects [225]. In another study, rats were treated for 6 weeks with nitro-L-arginine methyl ester to create hypertension in them. Then upon treatment of specially prepared olive leaves extract with a dose of 100 mg/Kg up to further 6 weeks, a normalization in blood pressure was observed [226]. An open study was conducted in Switzerland which included 40 borderline hypertensive monozygotic twins. One person from each pair was administered with 500 mg/day of olive leaf extract (EFLA 943) while the other was given a dose of 1000 mg/day. The treatment was carried out for 8 weeks. As a result it was noticed that blood pressure was reduced in all persons with more significant decrease in those treated with high dose. It was also noticed that cholesterol levels also decreased in all the treatments in a dose-dependent manner [227]. A commercial olive leaf extract (OLE) was investigated for its Ca^2+^ channel antagonistic effects on isolated rabbit hearts and cultured cardiomyocytes. OLE caused a decrease in systolic left ventricular pressure and heart rate as well as an increase in relative coronary flow in a concentration dependent fashion. In conclusion, OLE suppressed the L-type calcium channel directly and reversibly [228]. It was found that olive oil can significantly reduce the risk of stroke, heart attacks, stomach cancer, and other heart diseases [11]. Antihypertensive activities of* O. europaea* have also been summarized in Table 6.

### 4.7. Anti-Inflammatory and Antinociceptive Activities

Extra virgin olive oil has shown remarkable anti-inflammatory activity due to oleocanthal (**140**), a compound present in EVOO which has a strikingly similar profile to ibuprofen, a synthetic anti-inflammatory drug [229]. Studies on anti-inflammatory and antinociceptive effects of OLE on male Wistar rats showed that OLE doses of 50–200 mg/Kg produce dose-dependent analgesic effects and intraperitoneal administration of 200 mg/Kg OLE caused significant decrease in pain responses in formalin test [230]. The ethanolic and* n*-hexane extracts of olive fruits were investigated for their* in vivo* anti-inflammatory and antinociceptive activities. For anti-inflammatory activity the carrageenan-induced hind paw edema model was used and for the antinociceptive activity the hot plate test in mice was used. The results revealed that* n*-hexane extract showed activity at 400 mg/Kg dose while ethanolic extract did not show a significant activity [136]. A study was conducted to evaluate the traditional medicinal uses of olive leaves in Tunisia. Intraperitoneal administration of essential oil of* O. europaea* at doses of 100, 200, and 300 mg/Kg caused a significant reduction in acetic acid-induced abdominal constrictions and paw edema in mice [135]. Eidi et al. studied the anti-inflammatory and antinociceptive activities of olive oil to scientifically prove the use of olive oil as pain relieving in folk medicine. The antinociceptive activity was studied using hot plate, formalin, and writhing tests while acute anti-inflammatory effects of olive oil in mice were studied using xylene ear edema test [231]. Similar set of studies was also conducted to show the effects of maslinic acid (**104**) in acetic acid-induced writhing, inflammatory phase of formalin-induced pain, and capsaicin-induced mechanical allodynia in mice [232]. The results indicated that the olive oil only decreased the second phase of formalin-induced pain while it exhibited antinociceptive activity against writhing-induced pain in mice by acetic acid. In the xylene ear edema test, olive oil showed significant anti-inflammatory activity in the mice while maslinic acid also showed antiallodynic effects [231, 232]. Ursolic acid, a triterpenoid from olive leaves, has been reviewed for its anti- and proinflammatory activities [233]. Sahranavard et al. studied both the anti-inflammatory and antinociceptive activities of aqueous and methanolic extracts of defatted olive fruit. The results showed that both extracts did not exhibit good analgesic activity in the first phase of formalin test, whereas the methanolic and aqueous extracts inhibited the induced pain in the second phase of formalin test at doses of 600 mg/Kg and 450 mg/Kg, respectively [234]. Anti-inflammatory and antinociceptive activities of* O. europaea* have also been summarized in Table 7.

### 4.8. Gastroprotective Activities

A study was carried out to check the effect of olive leaf extract on the gastric defense system against experimentally induced gastric lesions by absolute ethanol in mice. The OLE was administered at a dose of 40, 80, and 120 mg/Kg while the reference drug ranitidine was given to the positive control at a dose of 50 mg/Kg, intragastrically. The protective effect of both the OLE and ranitidine was similar and in conclusion OLE possessed significant gastroprotective activity. It was suggested that the activity may be due to the antioxidants present in OLE [235]. Arsić et al. described the gastroprotective effect of olive oil extract in respect to its quercetin content. The quercetin content of the sample was confirmed by HPLC. Cold-restraint stress (CRS) induced rat gastric mucosa lesions test was applied to observe the gastroprotective effect of the sample. Olive oil extract showed gastroprotective activity close to quercetin, one of the most studied flavonoid with antiulcer properties [236]. Aqueous extract of the dried olive leaves was tested for its antiulcer effect in mice. Upon intragastric administration, it was found effective antiulcer agent against aspirin-induced gastric ulcers [237]. Olive fruit pulp (OFP) is a rich source of antioxidants and possesses very good hepatoprotective activity against CCl_4_-induced hepatic damage in mice [238].

### 4.9. Neuroprotective Activities

It has been reported that Mediterranean diet has a healthy effect on its people and they have a reduced risk of neurodegenerative [239] and cancer risks including breast and colon cancer [240, 241]. Guan et al. investigated the effect of maslinic acid (**104**), a triterpenoid isolated from olive leaves, on neuroprotection in type 2 diabetic rats. Streptozotocin was injected to induce neuronal death in mice. Maslinic acid showed a significant neuroprotective activity in a dose-dependent manner [242]. In another similar study, the effect of maslinic acid was observed on cultures of neurons from cerebral cortex. The results revealed that maslinic acid promoted a dose-dependent neuron survival during glutamate toxicity and may be a lead for natural neuroprotective drugs [243]. Qian et al. studied the effect of maslinic acid and its mechanism of action on cortical neurons using oxygen-glucose deprivation and then reoxygenation of 24 hours. Flow cytometry assay was used to monitor neuronal apoptosis and the results showed that maslinic acid alleviated the neuron injury in a dose-dependent manner [244].

The olive leaf extract was investigated for diabetic neuropathic pain on* in vitro* and* in vivo* models. Nerve growth factor (NGF) treated PC-12 cells were used for* in vitro* studies and streptozotocin-induced rat models were used for* in vivo* studies. Immunoblotting assay was used for the evaluation of neural apoptosis. The results showed that olive leaf extract decreased cell damage at a dose of 400 *μ*g/mL in NGF treated cells. OLE also showed potent DPPH radical scavenging activity [245]. Four groups of Wistar rats were studied for cerebral ischemia-reperfusion injury. One group was treated with distilled water and the other three groups were treated orally with OLE at doses of 50, 75, and 100 mg/Kg/day. According to the obtained results, OLE reduced infarct volume, blood-brain barrier permeability, brain edema, and improved neurologic deficit scores after transient middle cerebral artery occlusion (MCAO) [246]. In Alzheimer's disease, Tau aggregation into fibrillary tangles gives rise to intraneuronal lesions. Daccache et al. studied the ability of hydroxytyrosol (**84**), oleuropein (**1**), and oleuropein aglycone (**4**) isolated from olives to prevent* in vitro* tau fibrillization. Oleuropein was found to be even more active than the reference drug methylene blue on both wild-type and P301L Tau proteins at low micromolar concentrations [247]. OLE was studied for its effect on brain lipidomics in rat stroke model. OLE was administered to male Wistar rats at doses of 50, 75, and 100 mg/Kg/day. It increased the brain cholesterol ester, cholesterol, cerebroside, and phosphatidylcholine levels and reduced the brain ceramide levels on all doses while it increased the brain triglyceride levels only at doses of 75 and 100 mg/Kg/day [248].

Parkinson disease is the most common neurodegenerative syndrome resulting in slow death of midbrain dopaminergic neurons. Pasban-Aliabadi et al. designed a study to observe the effects of olive leaf extract and its main component oleuropein on 6-hydroxydopamine- (6-OHDA-) induced toxicity in rat adrenal pheochromocytoma (PC12) cells as an* in vitro* model of Parkinson's disease. Incubation of these cells with olive leaf extract (400 and 600 *μ*g/mL) and oleuropein (20 and 25 *μ*g/mL) decreased cell damage and reduced biochemical markers of cell death. Thus the results suggested the use of olive leaf extract and oleuropein in the treatment of Parkinson's disease as well [249]. Oleuropein aglycone (**4**) prevented the formation of toxic amyloid aggregates in brain, a characteristic feature of Alzheimer's disease [250].

### 4.10. Miscellaneous Activities

Diuretic activity of OLE has been studied through determination of natriuretic, saluretic, and carbonic anhydrase inhibition in lithium clearance experiments. According to the results, the aqueous methanolic and petroleum ether extracts of* O. europaea* possess efficient diuretic activity [251]. Wound healing activity of* n*-hexane and aqueous extract of dried olive leaves was observed using* in vivo* wound models in comparison with the reference drug Madecassol. The aqueous extract exhibited greater wound healing as well as antioxidant potential [252]. To study the antileech effects of olive methanolic extract, 100 leeches (*Limnatis nilotica*) were treated with methanolic extract of olives in comparison with the reference drugs Tinidazole and Levamisole. Tinidazole showed no effect on killing of leeches but Levamisole and olive extract killed leeches with a mean death time of 10 and 210 minutes, respectively [253]. Sato et al. studied antiallergic substances in olive waste materials of three Japanese varieties of* O*.* europaea* named as Mission, Lucca, and Manzanillo. The results showed that the antiallergic activity of 3,4-DHPEA-EA was greater than that of hydroxytyrosol and elenolic acid with an IC_50_ value of 33.5 ± 0.6 *μ*g/mL [254]. A recent study suggested that virgin olive oil (VOO) reduced hypoxia-reoxygenation injury in rat brain. In that study 60 male Wistar rats were used and the effect of VOO on brain lipidomics during stroke was studied. The results revealed that VOO pretreatment for 30 days decreased the brain ceramide levels in doses of 0.5 and 0.75 mL/Kg/day [255].

## 5. Toxicology

The effects of OLE on the hematology, biochemistry, kidney, and liver of Wistar male albino rats were determined to evaluate its toxicity profile. Total 30 rats were included in the study and fed on the OLE for 6 weeks. They were divided into five groups with group 1 being the control group (regular diet without OLE), and the other four groups fed on 0.2%, 0.4%, 0.7%, and 0.9%, respectively. The factors that were studied were lactate dehydrogenase (LDH), concentrations of alkaline phosphatase (ALP) in serum, cholesterol, bilirubin, triglycerides, and glucose levels. Groups 3, 4, and 5 showed a significant increase in the total bilirubin and serum ALP levels while cholesterol, glucose, and serum triglyceride levels were decreased in these groups as compared to the control group. Group 5 was affected the most as its liver and kidneys showed alterations in their tissues including necrosis of hepatocytes and a slight hemorrhage. So it was concluded that OLE should be used with care, especially, when being used at higher doses for longer periods of times as it may have undesirable effects on liver and kidneys [256]. Safety profile of maslinic acid (**67**), a compound isolated from the cuticle of* O. europaea*, was assessed by oral administration of high doses to mice. It was observed that a single oral administration of 1000 mg/Kg to mice did not produce any adverse effects and upon administration of a daily dose of 50 mg/Kg for 28 days did not produce any symptoms of toxicity [257].

## 6. Conclusion

The extensive literature survey revealed* O. europaea* to be a sacred and important medicinal plant used for the treatment of stomach problems, diabetes, hypertension, diarrhea, respiratory and urinary tract infections, skin diseases, bacterial and fungal infections, hemorrhoids, rheumatism, asthma, and hair loss. Pharmacological studies carried out on the fresh plant materials, crude extracts, and isolated components of* O. europaea* provide a reasonable support for its various traditional uses. Recent studies have been carried out focusing on evaluation of the antidiabetic, anticancer, antimicrobial, antifungal, antiviral, antioxidant, antihypertensive, gastroprotective, anti-inflammatory, antinociceptive, neuroprotective, and cardioprotective activities. Most of the mentioned pharmacological studies were aimed at confirming its traditional uses. It has been found that some of its traditional uses like antioxidant, antidiabetic, anticancer, and so forth have extensively been explored by several researchers. Most of the pharmacological studies carried out on* O. europaea* have been conducted on crude extracts of different parts of the plant and very few pharmacological reports are present for pure compounds isolated from the title plant of which oleuropein, maslinic acid, oleanolic acid, and so forth are the most abundant and notable. Thus it is somehow difficult to reproduce the results of all the studies and pinpoint the exact bioactive metabolites. Hence, there is a further need of studying the pure isolates for their pharmacological properties. Phytochemical research carried out on* O. europaea* has led to the isolation of many classes of compounds like iridoids, secoiridoids, lignans, biophenols, flavonoids, flavone glycosides, isochromans, and terpenoids, from less polar fractions but the polar fractions, that is,* n*-butanol and water soluble fractions, still possess a vast scope for further phytochemical studies. The outcome of already reported phytochemical studies should further expand its therapeutic potential for further pharmacological studies, especially* in vivo* studies. Hence some of the depicted interesting biological activities of* O. europaea* can be further proceeded to make use of them as a future therapy. The outcome of the future research in the above-mentioned areas will provide a convincing support for the future clinical use of* O. europaea* in modern medicine.

## Figures and Tables

**Table 1:** Common names of *Olea europaea*.

S. no.	Region	Name
1	Spain	Aceituna, Olivera, Olivo, Oliondo, Oastre, Oliba
2	Lithuania	Alyvos
3	Portugal	Azeitona, Oliveira
4	Romania	Culoare masline, Maslin, Masliniu, Oliva
5	Albania	Dege e ullirit, Ngjyre ulliri, Ulli
6	Greece	Elia
7	Estonia	Euroopa olipuu, Olipuus
8	Sweden	Oliv, Olivgront
9	Slovakia	Oliva europska
10	Czech Republic	Oliva, Olivove drevo
11	Latvia	Olivas
12	Denmark	Oliven
13	Germany	Olivenbaum, Olbaum
14	Netherlands	Olijf
15	Israel	Eylbert. Masline, Zayit
16	India	Jaitun, Julipe, Saidun
17	Armenia	Jitabdoogh, Jiteni
18	Bulgaria	Maslina
19	Croatia	Maslinov
20	South Africa	Mohlware
21	Hungary	Olajbogyo, Olajfa, Oliva
22	England	Olewydden, Olive
23	Iceland	Olifa
24	Finland	Oliivi, Oljypuu
25	Arab World	Zaitoon
26	France	Oulivie, Olive
27	Switzerland	Uliva
28	Pakistan	Zaitun
29	Tanzania	Zeituni
30	Georgia	Zetis

**Table 2:** Traditional and contemporary uses of *Olea europaea*.

S. no.	Part/preparation used	Ailment/use	Reference
1	Leaves and fruits/infusions and macerations	Hypoglycemic, hypotensive	[142]
2	Decoction or infusion of fruits and leaves	Antidiabetic	[140]
3	Olive oil + lemon juice	To treat gallstones	[139]
4	Oil of seeds/taken orally	Laxative	[118]
5	Decoctions of dried leaves and fruit/oral use	Diarrhea, respiratory, and urinary tract infections	[119]
6	Olive oil/applied on scalp	To prevent hair loss	[120]
7	Boiled extract of fresh leaves/taken orally	To treat asthma	[124]
8	Boiled extract of dried leaves/taken orally	To treat hypertension	[131, 258]
9	Leaves extract in hot water	Diuretic	[130]
10	Olive oil	Applied over fractured limbs	[132]
11	Infusion of leaves/oral use	Antipyretic	[127]
12	Olive fruit	Skin cleanser	[133]
13	Infusion of leaves/oral use	Anti-inflammatory, tonic	[258]
14	Leaf preparations	To treat gout	[134]
15	Leaves of *O*. *europaea *	Antibacterial	[135]
16	Decoction of leaves	Antidiabetic, antihypertensive	[138]
17	Fruits and leaves	Hemorrhoids, rheumatism, and vasodilator	[136]
18	Infusions of leaves	Eye infections treatment	[137]

**Table 3:** Antidiabetic activities of *Olea europaea*.

S. no.	Part/type of extract/compound	Disease/assay	Animal model	Effective dose	Reference drug	Reference
1	Olive leaf biophenols (oleuropein + hydroxytyrosol)	Insulin sensitivity improvement	Middle aged overweight men	58.8 mg	—	[151]
2	Aqueous extract of leaves	Antidiabetic assay	Streptozotocin induced diabetic rats	200 mg/Kg	Metformin	[259]
3	Oleanolic acid	Diabetic neuropathy prevention	Sprague Dawley rats	60 mg/Kg	Streptozotocin	[149]
4	Ethanolic extract of leaves	Antidiabetic activity	Male adult Wistar rats	0.5 g/Kg	Glibenclamide	[146]
5	Oleuropein and hydroxytyrosol	Antidiabetic and antioxidant activities	Adult male Wistar rats	16 mg/Kg	Trolox	[145]
6	Oleuropein	Hypoglycemic and antioxidant effect	Male New Zealand rabbits	20 mg/Kg	Alloxan	[143]
7	Oleanolic acid	Antihyperglycemic activity	*In vitro *	—	Oleuropein	[144]
8	Oleanolic acid demethyl	*α*-Amylase and lipase inhibition	*In vitro *	—	Oleuropein	[117]

**Table 4:** Anticancer activities of *Olea europaea*.

S. no.	Part/type of extract/compound	Cell line used	Activity	Reference drug	Reference
1	Maslinic acid	HT29 human colon cancer cell line	Dose dependent activity	Hydroxytyrosol	[161]
2	Hydroxytyrosol and hydroxytyrosyl laurate	Human monocytoid cell line	The best	H_2_O_2_	[158]
3	Oleanolic acid	Human hepatoma cell line HepG2	Good	—	[157]
4	Methanolic extract of leaves	Human leukemic cell line	High activity	5-Bromodeoxyuridine	[156]
5	Erythrodiol, uvaol, oleanolic acid, and maslinic acid	MCF-7 human breast cancer cell lines	High activity	Trolox	[155]
6	Water and methanolic extracts of olive leaves	Human breast adenocarcinoma (MCF-7), human urinary bladder carcinoma (T-24), and bovine brain capillary endothelial (BBCE)	Good	FGF-2	[154]
7	Aqueous extract of leaves	MKN45 (stomach cancer), MFC7 (breast cancer), NCI-H460 (lung cancer), HCT116 (colon cancer)	Good	MTT	[171]
8	Olive leaf extract	MKN45, HCT116, NCI-H460, and MCF7	Good	—	[171]
9	Erythrodiol	HT-29 human adenocarcinoma cells	Dose dependent activity	Caspase-3	[153]
10	Maslinic acid	HT29 human colon cancer cell line	Dose dependent activity	Hydroxytyrosol	[160]
11	Virgin olive oil phenolics extract	HT115 human colon cancer cells	Dose dependent activity	—	[260]
12	Oleuropein	HeLa human cervical carcinoma cells	Good	—	[261]

**Table 5:** Antioxidant and antimicrobial activities of *Olea europaea*.

S. no.	Part/type of extract/compound	Assay	Activity	Method/reference drug	Reference
1	Leaves extract	Antioxidant	High	Oxidation of soybean under microwaves	[213]
2	Olive leaves infusion	Antioxidant	Good	Hydroxyl and 2,2-diphenyl-1-picrylhydrazyl (DPPH) radicals	[196]
3	Ethanolic extract of leaves	Antioxidant	High	DPPH radicals	[197]
4	Olive fruit	Antioxidant	Good	DPPH radicals	[199]
5	Volatile fractions of leaves	Antibacterial and antifungal	Moderate to high	Microwell dilution assay/DMSO	[175]
6	Aqueous extract of leaves	Antimicrobial	Remarkable	Paper disc diffusion method/erythromycin	[174]
7	Acetone extract of leaves	Antibacterial	Good	Paper disc diffusion method/oleuropein	[172]
8	Fruit and leaves	Antioxidant	Good	DPPH radicals	[136]
9	Olive pulp	Antioxidant	Excellent	Vitamin C	[211]
10	Alperujo (olive waste)	Antimicrobial	Good	Syringic acid	[177]
11	Volatiles of olive fruits	Antibacterial and antifungal	Moderate	Amphotericin, levofloxacin	[181]
12	Aqueous extract of leaves	Antioxidant	Good	Trolox	[208]
13	Leaf extract	Antioxidant	Good	DPPH radicals	[200]
14	Aqueous solutions from table olives	Antibacterial and antifungal	Good	Streptomycin, oxytetracycline	[178]
15	Leaves extract	Antioxidative stress	Moderate	MTT assay/oleuropein	[147]
16	Maslinic acid	Antiparasitic	Dose dependent	Phenylmethanesulfonyl fluoride	[182]
17	Dry extract of table olives	Antioxidant	Very good	Trolox	[204]
18	Acetone extract of olive leaves + oleuropein	Antibacterial	Very good	Oleuropein	[172]
19	Leaf extract	Antioxidant and antimicrobial	Excellent	Oleuropein	[173]
20	Oleuropein aglycones, 3,4-DHPEA-EA, and 3,4-DHPEA-EDA	Anti-oxidative damage to erythrocytes	Good	Hydroxytyrosol	[207]
21	High strength leaf extract	Antimicrobial	Excellent	—	[170]
22	Dhokar cultivar olives	Antioxidant	Good	DPPH radicals	[201]
23	Aqueous leaf extract	Antioxidant and antimicrobial	Good	DPPH radicals/streptomycin	[171]
24	Leaf extract	Antioxidant and antimicrobial	Dose dependent	Ascorbic acid	[171]
25	Phenolics released by hydrothermal treatment of olive tree pruning	Antioxidant	Good	Trolox	[205]
26	Leaf extract	Antioxidant	Good	Trolox	[262]
27	Aqueous extract of leaves	Antifungal	The best	Oleuropein	[179]
28	Aqueous extract of leaves	Antimicrobial	Good	—	[167]
29	Dialdehydic form of decarboxymethyl elenolic acid	Antimicrobial	More potent than oleuropein	Hydroxytyrosol	[168]
30	Leaves sprayed with copper formulations	Antioxidant	Low activity due to decrease in phenolic content	*α*-Tocopherol and gallic acid	[206]
31	Aqueous ethanolic extract	Antioxidant activity	Good	*α*-Tocopherol and gallic acid	[198]
32	Aliphatic aldehydes from olive fruit	Antifungal and antielastase	Active except *Candida* spp.	Miconazole	[180]
33	Table olives from Portugal	Antioxidant and antimicrobial	Active except *Candida* spp.	*α*-Tocopherol, TBHQ, ampicillin, and cycloheximide	[263]
34	Extracts of leaves, fruits, and seeds	Antioxidant	High	Trolox	[202]
35	Oleuropein, hydroxytyrosol, and tyrosol	Antioxidant	High	Vitamin E, BHT	[185]
36	Long chain *α*, *β*-unsaturated aldehydes from olive fruit	Antimicrobial	Broad spectrum activity	—	[166]
37	Phenolics from olive oil mill waste water	Antioxidant	Good	2,3-Tert-butyl-4-hydroxyanisole, L-ascorbic acid	[192]
38	Maslinic acid	Antioxidant	Good	Silymarin	[193]
39	EVOO	Antioxidant	Good	Methyl linoleate	[191]
40	Phenolics	Antioxidant	Good	Ascorbic acid	[195]

**Table 6:** Antihypertensive activities of *Olea europaea*.

S. no.	Part/type of extract/compound	Assay	Activity	Reference
1	Uvaol, ursolic acid, and oleanolic acid	Cardiotonic	Good for uvaol and oleanolic acid	[219]
2	Leaf extract	Antihypertensive	Very good	[220]
3	Leaf extract	*In vivo* studies	Dose dependent	[221]
4	Leaves infusion	*In vivo* studies	Active on prolonged use	[223]
5	Oleanolic and ursolic acid	*In vivo* studies in insulin resistant rats	Active on 60 mg/Kg dose	[224]
6	Leaf extract (EFLA 943)	*In vivo* studies in rats	Active on 100 mg/Kg dose	[264]
7	Leaf extract (EFLA 943)	Studies in borderline hypertensive monozygotic twins	Good activity	[227]

**Table 7:** Anti-inflammatory and antinociceptive activities of *Olea europaea*.

S. no.	Part/type of extract/compound	Disease/assay	Animal model	Effective dose	Reference drug	Reference
1	Oleanolic acid	Antinociceptive	Female CD-1 mice (Barcelona, Spain)	—	Ibuprofen, pregabalin, baclofen	[232]
2	Olive oil	Antinociceptive and anti-inflammatory	Male Balb/C mice	10 mg/Kg	Dexamethasone	[231]
3	*n*-Hexane extract of fruits	Anti-inflammatory and antinociceptive	Male Swiss albino mice	400 mg/Kg	Indomethacin, acetylsalicylic acid	[136]
4	Olive oil	Analgesic and anti-inflammatory	Male Wistar rats	100–300 mg/Kg	Acetyl salicylate of lysine	[135]
5	*n*-Hexane extract of fruit	Anti-inflammatory and antinociceptive	Male Swiss albino mice	400 mg/Kg	Indomethacin, acetylsalicylic acid	[136]
6	Ethanolic extract of dry leaves	Antinociceptive, antihyperalgesic	Male Wistar rats	200 mg/Kg	Morphine	[230]
7	EVOO, Oleocanthal	Ibuprofen like activity	*In vitro *	—	Ibuprofen	[229]
